# Culprit vessel vs. immediate multivessel vs. out-of-hospital staged intervention for patients with non-ST-segment elevation myocardial infarction and multivessel disease

**DOI:** 10.3389/fcvm.2022.1033475

**Published:** 2022-11-23

**Authors:** Chen Wang, Jiachun Lang, Jingxia Zhang, Yuecheng Hu, Chuyi Han, Rongdi Xu, Jikun Wu, Chunwei Liu, Wenyu Li, Tingting Li, Ao Wei, Wei Qi, Dongxia Jin, Hongliang Cong, Le Wang

**Affiliations:** ^1^Clinical School of Thoracic, Tianjin Medical University, Tianjin, China; ^2^Department of Cardiology, Tianjin Chest Hospital, Tianjin, China

**Keywords:** non-ST-segment-elevation myocardial infarction, multivessel coronary disease, percutaneous coronary intervention, non-infarct-related artery, staged intervention

## Abstract

**Background and aims:**

The optimal interventional strategy remains undetermined in hemodynamically stable patients with NSTEMI and MVD. This study aimed to examine clinical prognosis among culprit vessel, immediate multivessel, and staged percutaneous coronary intervention (PCI) in patients with NSTEMI and MVD.

**Methods:**

This retrospective, observational, single-center study included 943 hemodynamically stable patients with NSTEMI and MVD who had undergone successful drug-eluting stent (DES) implantation from January 2014 to December 2019. Patients were categorized into culprit lesion-only PCI (CL-PCI), immediate multivessel PCI (MV-PCI), and out-of-hospital staged MV-PCI according to PCI strategy. The primary outcome was the composite of major adverse cardiac events (MACEs), including all-cause death, myocardial infarction (MI), or unplanned repeat revascularization. The secondary outcomes were all-cause death, cardiac death, MI, and unplanned repeat revascularization.

**Results:**

Over a median follow-up of 59 months, immediate MV-PCI was associated with a lower risk of all-cause death than CL-PCI (HR: 0.591, 95%CI: 0.364–0.960, *P* = 0.034). Out-of-hospital staged MV-PCI was associated with a reduced risk of MACE (HR: 0.448, 95%CI: 0.314–0.638, *P* < 0.001) and all-cause death (HR: 0.326, 95%CI: 0.183–0.584, *P* < 0.001) compared with CL-PCI. The above results were accordant after multivariate COX analysis and propensity score matching. MACE (HR: 0.560, 95%CI: 0.385–0.813, *P* = 0.002) and repeat revascularization (HR: 0.627, 95%CI: 0.400–0.982, *P* = 0.041) were significantly less likely to occur with out-of-hospital MV-PCI rather than immediate MV-PCI. However, the incidences of primary and secondary outcomes were comparable between immediate and staged PCI after confounder adjustment using multivariate regression and propensity score matching analysis. For subgroup analyses stratified by synergy between PCI with taxus and cardiac surgery score, staged MV-PCI was found to lower the risk of MACE compared with immediate MV-PCI in patients with more complex coronary disease.

**Conclusion:**

Hemodynamically stable patients with NSTEMI and MVD benefited from the strategy of MV-PCI. Patients with complex coronary anatomy treated with out-of-hospital staged MV-PCI rather than immediate MV-PCI had lower risks of MACE. These need to be confirmed in the future randomized study.

## Introduction

Despite remarkable advances in the prevention and treatment of coronary artery disease (CAD), the incidence of non-ST-segment-elevation myocardial infarction (NSTEMI) continues to rise (1). Compared to ST-segment-elevation myocardial infarction (STEMI), NSTEMI is prone to have a higher risk of death after discharge (2, 3). Multivessel disease (MVD) is found in up to 40–70% of patients presenting with NSTEMI (4, 5). Patients with NSTEMI and MVD are associated with poorer clinical prognosis than those with single-vessel disease (6, 7).

Percutaneous coronary intervention (PCI) is the prevalent revascularization strategy for NSTEMI patients (8). However, the optimal interventional strategy for NSTEMI and MVD remains unclear. Patients may undergo the following three interventional strategies: (1) culprit lesion intervention only at the index PCI, (2) multivessel intervention during index procedure, or (3) staged multivessel intervention after discharge or during index admission. Recent guidelines only provide class II recommendations and level B evidence for immediate multivessel percutaneous coronary intervention (MV-PCI) in NSTEMI with MVD (9). Obviously, controversy and uncertainty remain regarding the superiority of MV-PCI during the index procedure and whether patients with NSTEMI and MVD may benefit from staged MV-PCI. Most previous studies have been limited to comparisons of in-hospital staged MV-PCI and immediate MV-PCI (10, 11). Data comparing out-of-hospital and immediate MV-PCI are scarce. Stent generation was found to be a considerable factor of major adverse cardiac events (MACEs) in NSTEMI with MVD (12). In contemporary PCI practice, second-generation drug-eluting stent (DES) has replaced first-generation DES. Therefore, the object of our study was to assess clinical prognosis between culprit-only PCI (CL-PCI), immediate MV-PCI, and out-of-hospital staged MV-PCI using a Chinese single-center database of NSTEMI patients with MVD who received newer-generation DESs during procedure to elucidate the optimal interventional method for NSTEMI patients with MVD.

## Materials and methods

### Study population and definitions

This was a single-center observational retrospective study of consecutive patients presenting with NSTEMI with MVD. All patients enrolled in the present study have undergone successful PCI between January 2014 and December 2019 at Tianjin Chest Hospital (Tianjin, China) and tolerated dual anti-platelet therapy for 12 months or more. The exclusion criteria were as follows: STEMI, single-vessel disease, cardiogenic shock, failed PCI, death within 2 months after discharge, patients who received staged MV-PCI during the primary admission, staged PCI beyond sixty days, prior coronary artery bypass grafting (CABG), CABG within 60 days after primary admission, and loss to follow-up. The study protocol was approved by the Ethics Committee of Tianjin Chest Hospital and followed the principles of the Declaration of Helsinki. Written informed consent was waived as the data used in this study were anonymous and retrospective. According to the interventional strategy, the participants were categorized into three groups: CL-PCI group, Immediate MV-PCI group, and staged out-of-hospital MV-PCI group. CL-PCI was defined as a PCI only for culprit lesion performed. Immediate MV-PCI was defined as a PCI for both non-infarct-related artery (IRA) and IRA performed during the index procedure. Staged MV-PCI was defined as staged PCI for non-IRA within 60 days (13–17). The interval was selected to avoid losing patients who received the second procedure for more than sixty days after index procedure.

Our definition of NSTEMI adheres to the 4th universal definition of MI (18). MVD was defined as the occurrence of ≥ 70% stenosis of ≥ two major epicardial arteries or ≥ 50% stenosis of the left main coronary artery. Successful procedure was defined as visually estimated residual luminal stenosis of < 30% ultimately, followed by thrombolysis in myocardial infarction grade III flow. We used the residual SYNTAX score to quantify complete revascularization (CR). Angiographic CR was defined as a residual SYNTAX score of 0 (19–21). All SYNTAX scores were quantified by experienced independent analysts at Tianjin Chest Hospital.

### Percutaneous coronary intervention procedure and medical treatment

All procedures were performed according to current guidelines. Before PCI, loading doses of anti-platelet drugs were prescribed to all patients except those who received these drugs as regular therapy. Interventional strategies, timing of MV-PCI, stent type, and stent technology were selected at the discretion of interventional cardiologists. Identification of the culprit lesion conducted by interventional operators according to each patient’s electrocardiogram, non-invasive imaging, angiographic imaging, and anatomic imaging. All patients were required to be on dual anti-platelet therapy for 12 months or more after procedure. Patients were clinically followed-up *via* telephone interviews or outpatient visits.

### Clinical outcomes

A composite of MACE was the primary outcome defined as all-cause death, myocardial infarction (MI), or any repeat revascularization. All-cause death, cardiac death, MI, and any repeat revascularization were prespecified as the secondary outcomes. The definition of all-cause death was death from any cause. Diagnosis of MI required clinical evidence of acute myocardial ischemia and increased levels of cardiac-specific biomarkers, angina symptoms, specific changes in electrocardiograms, or imaging evidence. Repeat revascularization was defined as any revascularization driven by clinically or angiographically needs based on Academic Research Consortium definitions.

### Statistical analysis

All analyses were conducted using SPSS software version 26.0. Continuous variables were presented as mean ± *SD* or as medians [interquartile range (IQR)] according to normally distribution. The Kolmogorov-Smirnov test was performed to assess normality of distribution. Normally distributed data were compared using one-way ANOVA or Kruskal-Wallis H test for multigroup analyses, as appropriate. Categorical variables were reported as counts and percentages, and comparisons were examined using Pearson’s chi-squared test or Fisher’s exact test. Cumulative incidences of the clinical outcomes between the groups stratified by revascularization strategy were estimated using the Kaplan-Meier curve, and comparisons were calculated with the log-rank test. To assess the risk of clinical outcomes, multivariable Cox proportional hazards regression analysis was performed to offer hazard ratios (HRs) with 95% confidence intervals (CIs). We performed sensitivity analyses to adjusted for confounders to minimize the effects of significantly different baseline characteristics. First, variables associated with clinical outcomes were selected using univariate Cox proportional hazards analysis. Clinically meaningful reference values were used to dichotomize continuous variables. Variables with a *P*-value < 0.1 were added to multivariable Cox regression models. Second, we computed propensity score matching between the groups by performing a logistic regression analysis with 27 baseline variables. Patients between groups were matched in a 1:1 manner using a greedy matching strategy with calipers (Supplementary Tables 1 through 6). Subgroup analyses were conducted by age, presence of diabetes mellitus, triple vessel disease, Global Registry of Acute Coronary Events (GRACE) score, and synergy between PCI with taxus and cardiac surgery (SYNTAX) score to compare primary endpoints among the three interventional strategies and assess the interactions between these covariates and clinical outcomes. A two-tailed *P* of < 0.05 indicated statistical significance.

## Results

### Baseline characteristics

**FIGURE 1:**
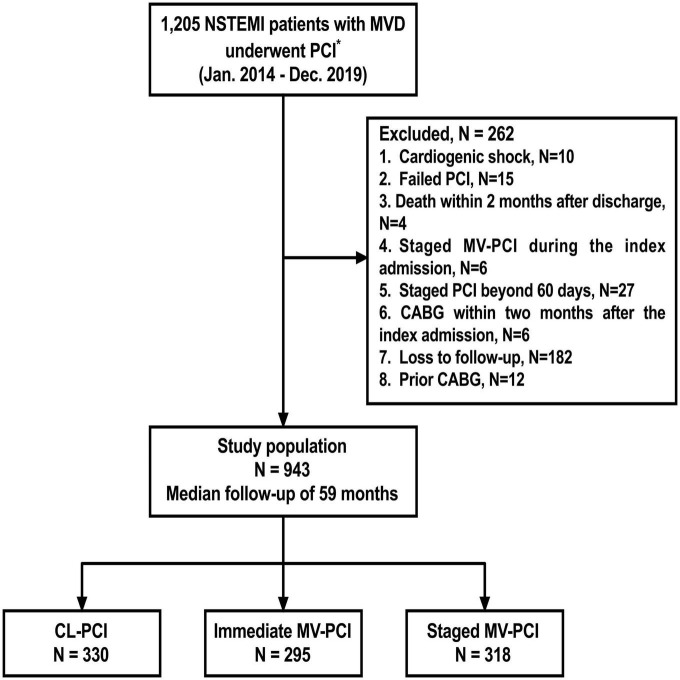
Study flowchart. *All patients could tolerate dual anti-platelet therapy for at least 12 months. NSTEMI, non–ST-segment–elevation myocardial infarction; MVD, multivessel disease; PCI, percutaneous coronary intervention; MV-PCI, multivessel percutaneous coronary intervention; CL-PCI, culprit-only percutaneous coronary intervention; CABG, coronary artery bypass grafting.

We analyzed 943 consecutive MVD patients treated with PCI for NSTEMI. The median follow-up duration was 59 months. Flow chart is presented in Figure 1. Of these, 330 patients (35.0%) underwent CL-PCI, 295 patients (31.3%) underwent immediate MV-PCI, and 318 (33.7%) underwent staged MV-PCI. Tables 1, 2 summarize clinical, angiography, and procedures characteristics of patients (baseline characteristics after propensity score matching are described in Supplementary Tables 1–6). The immediate MV-PCI group had a higher GRACE score [123.0 (105.8–146.0) vs. 126.0 (108.0–147.0) vs. 122.0 (102.0–139.0), *P* = 0.028]. They also tended to have a higher proportion of ST-segment depression and a higher heart rate. The CL-PCI group more often had estimated glomerular filtration rate (eGFR) < 60 ml/min/1.73 m^2^ than the other two groups. Additionally, the CL-PCI group had a lower percentage of aspirin treatment among the three groups (97.3% vs. 99.7% vs. 98.7%, *P* = 0.044). Except for aspirin, no differences in medications at discharge were observed among the groups. A significant difference of culprit lesion locations among the groups was found. For overall PCI procedures, patients receiving staged MV-PCI had a higher prevalence of type B_2_/C lesions, a higher rate of glycoprotein IIb/IIIa inhibitor treatment, a greater total numbers of stents, longer stent length, and longer total hospital length of stay. Patients of the staged PCI group underwent the second-stage procedure at a median of 22.5 (IQR: 18.0–29.0) days after the index PCI. In addition, the minimum luminal diameter was notably longer in the CL-PCI group, whereas the proportion of left main disease was higher in the patients who underwent immediate MV-PCI.

**TABLE 1:** Baseline clinical characteristics.

	CL-PCI (*N* = 330)	Immediate MV-PCI (*N* = 295)	Staged MV-PCI (*N* = 318)	*P-value*
Age, y	64.0 (57.8–72.0)	64.0 (57.0–71.0)	63.0 (57.0–69.0)	0.154
>65 years	148 (44.8%)	125 (42.4%)	124 (39.0%)	0.318
Male	235 (71.2%)	219 (74.2%)	234 (73.6%)	0.664
Killip class II–III	26 (7.9%)	25 (8.5%)	13 (4.1%)	0.060
GRACE score	123.0 (105.8–146.0)	126.0 (108.0–147.0)	122.0 (102.0–139.0)	0.028
>140	98 (29.7%)	92 (31.2%)	71 (22.3%)	0.030
ST-segment depression	101 (30.6%)	111 (37.6%)	81 (25.5%)	0.005
**Past medical history**				
Hypertension	234 (70.9%)	204 (69.4%)	206 (64.8%)	0.221
Diabetes mellitus	111 (33.6%)	113 (38.3%)	121 (38.1%)	0.385
Prior MI	33 (10.0%)	31 (10.5%)	32 (10.1%)	0.975
Prior PCI	41 (12.4%)	33 (11.2%)	25 (7.9%)	0.148
Prior stroke	61 (18.5%)	64 (21.7%)	54 (17.0%)	0.318
Current smoker	148 (44.8%)	139 (47.3%)	157 (49.4%)	0.514
LVEF, %	55.0 (50.0–60.0)	56.0 (48.0–59.0)	56.0 (51.0–59.0)	0.124
< 50%	79 (23.9%)	80 (27.1%)	60 (18.9%)	0.050
SBP, mmHg	131.0 (120.0–143.5)	132.0 (118.0–146.0)	132.0 (120.0–144.0)	0.878
HR, bpm	69.0 (60.0–78.0)	71.0 (63.0–80.0)	69.5 (61.0–78.0)	0.028
**Laboratory findings**				
Peak level of troponin, ng/ml	0.6 (0.2–1.2)	0.5 (0.2–1.1)	0.6 (0.3–1.3)	0.169
Peak level of CK-MB, ng/ml	28.0 (17.0–60.0)	30.0 (17.0–53.6)	33.0 (19.0–61.3)	0.098
eGFR, ml/min/1.73 m^2^	86.3 ± 28.3	87.6 ± 25.8	92.4 ± 24.6	0.009
< 60 ml/min/1.73 m^2^	52 (15.8%)	37 (12.5%)	24 (7.5%)	0.005
**Medications at discharge**				
Aspirin	321 (97.3%)	294 (99.7%)	314 (98.7%)	0.044
P_2_Y_12_ inhibitor	330 (100.0%)	295 (100.0%)	318 (100%)	NA
Clopidogrel	275 (83.3%)	226 (76.6%)	264 (83.0%)	–
Ticagrelor	55 (16.7%)	69 (23.4%)	54 (17.0%)	–
ACEI or ARB or ARNI	206 (62.4%)	198 (67.1%)	200 (62.9%)	0.413
Beta-blocker	225 (68.2%)	223 (75.6%)	234 (73.6%)	0.097
Statin	321 (97.3%)	277 (93.9%)	302 (95.0%)	0.115

GRACE, Global Registry of Acute Coronary Events; MI, myocardial infarction; PCI, percutaneous coronary intervention; LVEF, left ventricular ejection fraction; SBP, systolic blood pressure; HR, heart rate; CK-MB, creatine kinase-myocardial band; eGFR, estimated glomerular filtration rate; ACEI, angiotensin-converting enzyme inhibitor; ARB, angiotensin-II receptor blocker; ARNI, angiotensin receptor neprilysin inhibitor.

**TABLE 2:** Coronary angiographic and procedural characteristics.

	CL-PCI (*N* = 330)	Immediate MV-PCI (*N* = 295)	Staged MV-PCI (*N* = 318)	*P-value*
**Culprit lesion profiles**				
Location of culprit lesions				<0.001
Left main coronary artery	12 (3.6%)	21 (7.1%)	4 (1.3%)	–
Left anterior descending artery	113 (34.2%)	124 (42.0%)	88 (27.7%)	–
Left circumflex artery	111 (33.6%)	108 (36.7%)	114 (35.8%)	–
Right coronary artery	110 (33.3%)	60 (20.3%)	146 (45.9%)	–
**ACC/AHA lesion type**				
B_2_/C	300 (90.9%)	246 (83.4%)	308 (96.9%)	<0.001
**Overall-lesion profiles**				
Left main disease	34 (10.3%)	42 (14.2%)	21 (6.6%)	0.008
Triple vessel disease	245 (74.2%)	208 (71.0%)	249 (78.3%)	0.114
SYNTAX score > 22	88 (26.7%)	67 (22.7%)	91 (28.6%)	0.240
**Procedural characteristics**				
Radial artery access	255 (77.3%)	232 (78.6%)	262 (82.4%)	0.252
Use of glycoprotein IIb/IIIa inhibitor	177 (53.6%)	141 (47.8%)	220 (69.2%)	<0.001
Total number of stents	1 (1–2)	2 (2–3)	3 (3–4)	<0.001
Minimum luminal diameter (mm)	2.8 (2.5–3.0)	2.5 (2.5–2.8)	2.5 (2.5–2.8)	<0.001
<3 mm	174 (52.7%)	227 (76.9%)	257 (80.8%)	<0.001
Total stent length > 30 mm	201 (60.9%)	266 (90.2%)	313 (98.4%)	<0.001
IVUS guide PCI	7 (2.1%)	7 (2.4%)	5 (1.6%)	0.769
IABP	8 (2.4%)	8 (2.7%)	4 (1.3%)	0.410
Complete revascularization	–	151 (51.2%)	198 (62.3%)	–
Interval between index and second stage PCI, d	–	–	22.5 (18.0–29.0)	–
Length of hospital stay, d	7.0 (5.0–8.0)	7.0 (6.0–9.0)	12.0 (10.0–14.0)	<0.001

ACC/AHA, American College of Cardiology/American Heart Association; SYNTAX, synergy between PCI with taxus and cardiac surgery; IVUS, intravascular ultrasound; PCI, percutaneous coronary intervention; IABP, intra-aortic balloon pump.

### Clinical outcomes of unadjusted populations

**FIGURE 2:**
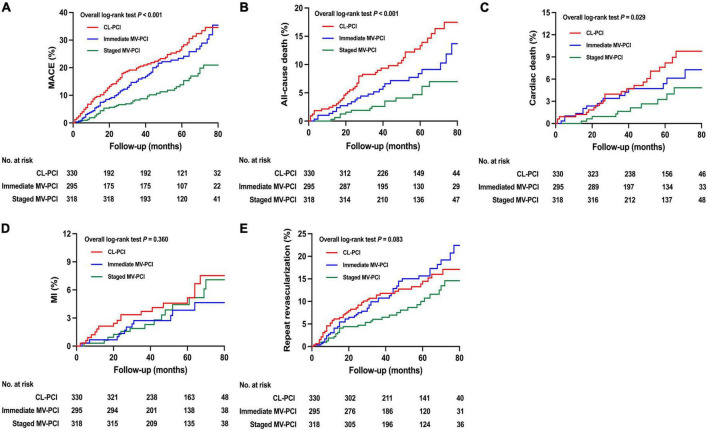
Unadjusted clinical outcomes. Cumulative incidence of **(A)** MACE, **(B)** all-cause death, **(C)** cardiac death, **(D)** MI, and **(E)** repeat revascularization. MACE, major adverse cardiac events; MI, myocardial infarction; CL-PCI, culprit-only percutaneous coronary intervention; MV-PCI, multivessel percutaneous coronary intervention.

The unadjusted primary and secondary clinical endpoints are presented in Table 3 and Figure 2, respectively. The CL-PCI group was inferior to the staged MV-PCI group for reducing MACE (HR: 0.448, 95%CI: 0.314–0.638, *P* < 0.001), in large part due to an increased risk of all-cause death and cardiac death. The incidence of all-cause death was higher in the CL-PCI group than in the immediate MV-PCI group (HR: 0.591, 95%CI: 0.364–0.960, *P* = 0.034). The staged strategy had a reduced incidence of MACE than the immediate multivessel strategy (HR: 0.560, 95%CI: 0.385–0.813, *P* = 0.002), mainly caused by a reduced risk of repeat revascularization.

**TABLE 3:** Clinical outcomes over entire follow-up period.

	CL-PCI (*N* = 330)	Immediate MV-PCI (*N* = 295)	Unadjusted HR (95%CI)	*P-value*	Adjusted HR (95%CI)	*P-value*	PS-matched HR (95%CI)	PS-matched *P-value*
MACE	96 (29.1%)	71 (24.1%)	0.793 (0.584–1.078)	0.139	0.759 (0.556–1.037)	0.083	0.720 (0.504–1.029)	0.071
All-cause death	47 (14.2%)	25 (8.5%)	0.591 (0.364–0.960)	0.034	0.544 (0.331–0.895)	0.017	0.493 (0.280–0.868)	0.014
Cardiac death	27 (8.2%)	16 (5.4%)	0.671 (0.361–1.245)	0.205	0.584 (0.307–1.110)	0.101	0.619 (0.292–1.311)	0.210
MI	21 (6.4%)	11 (3.7%)	0.586 (0.282–1.215)	0.151	0.553 (0.264–1.158)	0.116	0.570 (0.239–1.360)	0.206
Repeat revascularization	50 (15.2%)	47 (15.9%)	1.038 (0.697–1.546)	0.855	1.039 (0.693–1.559)	0.853	1.045 (0.654–1.670)	0.854

	**CL-PCI (*N* = 330)**	**Staged MV-PCI (*N* = 318)**	**Unadjusted HR (95%CI)**	***P-value***	**Adjusted HR (95%CI)**	***P-value***	**PS-matched HR (95%CI)**	**PS-matched *P-value***

MACE	96 (29.1%)	45 (14.2%)	0.448 (0.314–0.638)	<0.001	0.508 (0.352–0.733)	<0.001	0.501 (0.336–0.747)	0.001
All-cause death	47 (14.2%)	15 (4.7%)	0.326 (0.183–0.584)	<0.001	0.431 (0.235–0.792)	0.007	0.317 (0.155–0.646)	0.002
Cardiac death	27 (8.2%)	10 (3.1%)	0.392 (0.190–0.810)	0.011	0.542 (0.253–1.160)	0.115	0.357 (0.141–0.907)	0.030
MI	21 (6.4%)	16 (5.0%)	0.817 (0.426–1.567)	0.543	0.945 (0.480–1.860)	0.870	1.205 (0.573–2.533)	0.624
Repeat revascularization	50 (15.2%)	32 (10.1%)	0.651 (0.418–1.015)	0.058	0.647 (0.410–1.021)	0.061	0.702 (0.428–1.150)	0.160

	**Immediate MV-PCI (*N* = 295)**	**Staged MV-PCI (*N* = 318)**	**Unadjusted HR (95%CI)**	***P-value***	**Adjusted HR (95%CI)**	***P-value***	**PS-matched HR (95%CI)**	**PS-matched *P-value***

MACE	71 (24.1%)	45 (14.2%)	0.560 (0.385–0.813)	0.002	0.708 (0.474–1.056)	0.091	0.646 (0.405–1.031)	0.067
All-cause death	25 (8.5%)	15 (4.7%)	0.551 (0.291–1.046)	0.068	0.787 (0.391–1.582)	0.501	0.476 (0.204–1.113)	0.087
Cardiac death	16 (5.4%)	10 (3.1%)	0.581 (0.264–1.281)	0.178	0.890 (0.374–2.115)	0.791	0.353 (0.112–1.107)	0.074
MI	11 (3.7%)	16 (5.0%)	1.382 (0.641–2.979)	0.409	2.026 (0.825–4.975)	0.123	1.620 (0.589–4.460)	0.350
Repeat revascularization	47 (15.9%)	32 (10.1%)	0.627 (0.400–0.982)	0.041	0.723 (0.447–1.170)	0.187	0.763 (0.441–1.318)	0.332

MACCE, major adverse cardiac or cerebrovascular events; MI, myocardial infarction.

**FIGURE 3:**
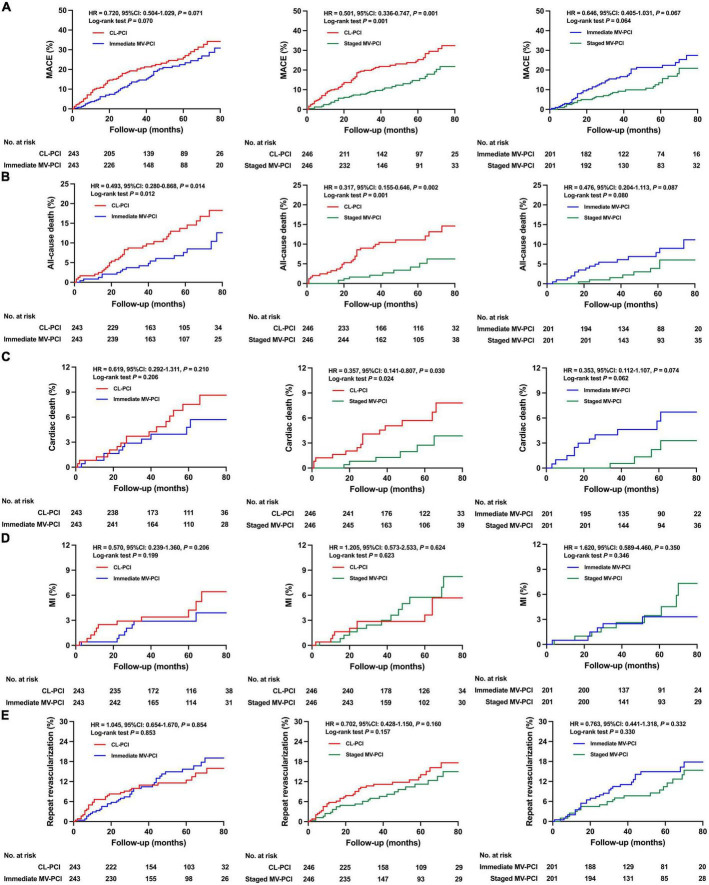
Clinical outcomes for propensity score matched patients. Cumulative incidence of **(A)** MACE, **(B)** all-cause death, **(C)** cardiac death, **(D)** MI, and **(E)** repeat revascularization. MACE, major adverse cardiac events; MI, myocardial infarction; CL-PCI, culprit-only percutaneous coronary intervention; MV-PCI, multivessel percutaneous coronary intervention.

The risk of all-cause death was notably lower in the immediate MV-PCI group than in the CL-PCI group after multivariate Cox analysis and propensity-score matching, as shown in Table 3 and Figure 3. The staged MV-PCI group showed a lower incidence of MACE, all-cause death, and cardiac death than the CL-PCI group in the propensity-score matching population. The results of the clinical endpoints between staged MV-PCI and CL-PCI in the multivariate Cox regression analysis also showed consistent results, except for a comparable risk of cardiac death. The study revealed a trend favoring the staged interventional strategy in terms of MACE compared with immediate interventional strategy, although there was no significant difference after various sensitivity analyses. Further details are presented in Table 3 and Figure 3.

### Independent predictors for major adverse cardiac event and all-cause death

Independent predictors for MACE and all-cause death identified using the multivariate Cox regression models were shown in Table 4. Diabetes mellitus, a history of PCI, and eGFR < 60 ml/min/1.73 m^2^ were independent predictors of MACE, while age > 65 years, diabetes mellitus, LVEF < 50%, and eGFR < 60 ml/min/1.73 m^2^ were independent predictors of all-cause death. Multistage MV-PCI was independently associated with a reduced risk of MACE (HR: 0.678, 95%CI: 0.461–0.997, *P* = 0.048), and MV-PCI was independently associated with a reduced incidence of all-cause death (HR: 0.579, 95% CI: 0.356–0.943, *P* = 0.028).

**TABLE 4:** Independent predictors of clinical outcomes.

	Univariate analysis		Multivariate analysis	
		
MACE	HR (95%CI)	*P-value*	HR (95%CI)	*P-value*
Age > 65 years	1.307 (0.998–1.711)	0.052	1.130 (0.851–1.499)	0.398
Killip class II-III	1.883 (1.249–2.839)	0.003	1.309 (0.836–2.048)	0.239
Diabetes mellitus	1.589 (1.213–2.081)	0.001	1.537 (1.164–2.028)	0.002
Prior MI	1.723 (1.189–2.499)	0.004	1.223 (0.805–1.860)	0.345
Prior PCI	2.193 (1.548–3.107)	<0.001	1.629 (1.097–2.419)	0.015
LVEF < 50%	1.428 (1.062–1.920)	0.018	1.078 (0.786–1.480)	0.640
eGFR < 60 ml/ min/1.73 m^2^	2.007 (1.427–2.824)	<0.001	1.475 (1.019–2.135)	0.040
ACC/AHA lesion type B_2_/C	0.688 (0.460–1.031)	0.070	0.708 (0.466–1.075)	0.105
Left main disease	1.865 (1.286–2.704)	0.001	1.443 (0.958–2.172)	0.079
Radial artery access	0.615 (0.458–0.826)	0.001	0.772 (0.560–1.065)	0.115
IABP	1.909 (0.898–4.054)	0.093	1.266 (0.539–2.973)	0.589
Multivessel MVR	0.609 (0.465–0.798)	<0.001	0.748 (0.547–1.024)	0.070
Multistage MVR	0.495 (0.356–0.688)	<0.001	0.678 (0.461-0.997)	0.048
**All-cause death**				
Age > 65 years	3.891 (2.431–6.227)	<0.001	2.915 (1.783-4.765)	<0.001
Killip class II-III	2.788 (1.598–4.866)	<0.001	1.286 (0.703–2.353)	0.414
Diabetes mellitus	1.629 (1.069–2.481)	0.023	1.573 (1.022–2.420)	0.039
Prior MI	2.064 (1.200–3.552)	0.009	1.516 (0.860–2.671)	0.150
LVEF < 50%	2.725 (1.783–4.165)	<0.001	1.715 (1.091–2.697)	0.019
eGFR < 60 ml/ min/1.73 m^2^	4.692 (3.010–7.314)	<0.001	2.393 (1.475–3.883)	<0.001
Radial artery access	0.483 (0.312–0.748)	0.001	0.703 (0.442–1.118)	0.137
IABP	2.520 (0.924-6.875)	0.071	1.764 (0.595-5.234)	0.306
Multivessel MVR	0.453 (0.297–0.690)	<0.001	0.579 (0.356–0.943)	0.028
Multistage MVR	0.404 (0.231–0.704)	0.001	0.710 (0.371–1.359)	0.301

GRACE, Global Registry of Acute Coronary Events; MI, myocardial infarction; PCI, percutaneous coronary intervention; LVEF, left ventricular ejection fraction; eGFR, estimated glomerular filtration rate; ACC/AHA, American College of Cardiology/American Heart Association; IABP, intra-aortic balloon pump; MVR, multivessel revascularization.

### Subgroup analysis

**FIGURE 4:**
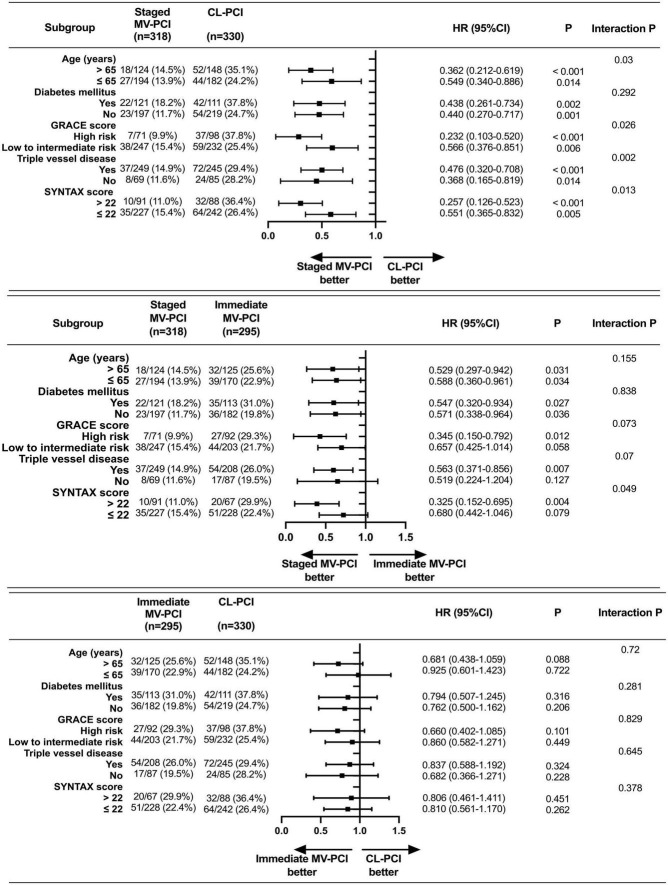
Subgroup analysis on MACE. MACE, major adverse cardiac events; eGFR, estimated glomerular filtration rate; LVEF, left ventricular ejection fraction; GRACE, Global Registry of Acute Coronary Events; SYNTAX, Synergy between PCI with Taxus and Cardiac Surgery. High risk: GRACE score > 140; Low to intermediate risk: GRACE score ≤ 140.

By contrast with the CL-PCI group in terms of MACE, the superiorities of staged MV-PCI were found across various subgroups, except for the presence of diabetes mellitus, for which a trend toward an intervention strategy-by-subgroup interaction was shown (Figure 4). However, we found a trend favoring staged PCI over immediate PCI in patients with whether age > 65 years or not, whether diabetes mellitus or not, high-risk (GRACE score > 140), or complex coronary disease (triple vessel disease), although there were no statistically significant interactions between these subgroups and the effects of interventional method for MACE. Among patients with SYNTAX score > 22, staged PCI was superior to immediate PCI for reducing MACE with a significant interaction. There were no significant interactions between subgroup factors and the effects of immediate MV-PCI relative to CL-PCI on MACE risks. Moreover, the risks of CL-PCI relative to immediate MV-PCI in terms of MACE were neutral in all subgroups.

## Discussion

The optimal management strategy for non-IRA continues to be debated in patients with NSTEMI and MVD who are undergoing PCI. The present study compared three interventional revascularization strategies (CL-PCI, immediate MV-PCI, and out-of-hospital staged MV-PCI) over the currently known longest reported follow-up period, and is the first known study to examine the efficacy of out-of-hospital staged PCI vs. immediate PCI in hemodynamically stable patients with NSTEMI and MVD. The present study has three main findings. First, immediate PCI was superior to CL-PCI for reducing all-cause death. Moreover, out-of-hospital staged MV-PCI also resulted in lower rate of MACE and all-cause death than CL-PCI. Second, long-term clinical outcomes were comparable between out-of-hospital staged and immediate MV-PCI after adjustment. Nevertheless, staged PCI was found to be superior to immediate PCI for reducing MACE in complex coronary disease (i.e., SYNTAX score > 22). Third, staged PCI was the independent predictor of reduced MACE, and multivessel interventional strategy was the independent predictor of reduced all-cause mortality. These findings offer meaningful insights into the management of hemodynamically stable patients with NSTEMI and MVD who are undergoing DESs implantation.

Despite the apparent consensus in guidelines on an early invasive approach for high-risk patients with NSTEMI, the optimal strategy for non-IRA among patients with hemodynamically stable NSTEMI remains unclear (9). MVD is common in patients with NSTEMI, and confers adverse prognosis (6, 7). Vulnerable plaques may occur in both IRAs and non-IRAs (22). Moreover, achieving accurate identification of culprit lesions is more challenging in NSTEMI, and performing a CL-PCI may lead to unwittingly intervention of a non-culprit lesion instead of an inapparent culprit lesion (22). Thus, revascularization in non-IRAs may further reduce recurrent incidences of ischemic events and obviate the requirement for unexpected revascularization. However, there remain several potential drawbacks to the MV-PCI strategy for intervention in non-IRAs. Multivessel PCI approach may potentially lead to increased procedure duration, radiation exposure, contrast volume, and in-hospital expenses (23, 24). Furthermore, assessment of non-culprit lesion stenosis severity based on a visual angiographic procedure is difficult during the acute setting, which may result in significant exaggeration of stenosis severity (25). Adopting complex multivessel revascularization may result in propensity for peri-procedural MI (23). Multivessel revascularization, especially one-time CR, has a high likelihood of increased stent thrombosis and inflammatory burden (26). Therefore, determining the ideal revascularization strategy for non-IRAs in patients with hemodynamically stable NSTEMI and MVD remains challenging.

Observational studies and prior meta-analyses comparing culprit lesion-only intervention with multivessel intervention in patients with NSTEMI and MVD have produced conflicting findings, with several recent studies showing significantly improved clinical benefits with MV-PCI (27–29). A large contemporary meta-analysis of 12 studies which included 117,685 patients with non-ST-segment elevation acute coronary syndrome (NSTE-ACS) demonstrated that multivessel strategy was not superior to intervention of IRA only (27). However, a multi-site observational registry of a propensity-matched population of 21,857 NSTEMI participants with MVD across London, UK, found that those treated with single-stage MV-PCI experienced a more favorable long-term outcome than those treated with CL-PCI (28). A recent South Korean trial found that multivessel strategy was superior to single-vessel strategy in lowering in-hospital mortality (MV-PCI vs. CL-PCI: 1.4% vs. 2.9%, *P* = 0.025) (29). This benefit with MV-PCI persisted concerning MACEs, mortality, or MI during the 1-year follow-up. In our study, multivessel strategy had better clinical prognosis than CL-PCI. We have found that the staged intervention approach was associated with reduced rates of MACE, all-cause death, and cardiac death when compared with the CL-PCI strategy in the propensity-matched analysis. Two sensitivity analyses demonstrated the superiority of the immediate MV-PCI strategy for all-cause death over the CL-PCI strategy. Additionally, multivessel revascularization was an independent predictor of all-cause death. Collectively, these findings suggest that MV-PCI leads to better clinical outcomes, which is in line with previous observational studies.

Although MV-PCI is considered a reasonable option for NSTEMI patients with MVD, little is known about the timing to perform intervention of non-IRAs in such patients. Several previous observational studies comparing the one-session strategy and staged-session strategy among patients with NSTE-ACS or stable CAD demonstrated similar clinical outcomes (30, 31). An American study showed comparable 3-year mortality incidences between the immediate strategy and staged strategy after propensity score matching in MVD patients without STEMI (30). Toyota et al. found no differences in 5-year and 30-day incidences of MACE (a combination of all-cause death/MI/stroke) between the immediate intervention and the staged intervention in patients with stable CAD or NSTE-ACS (31). However, two subgroup analyses demonstrated a survival benefit of staged PCI over immediate PCI for patients with NSTE-ACS (13, 14). Yu et al. reported that staged intervention strategy for intermediate-to high-risk NSTE-ACS patients was associated with reduced cardiac death or MI events at 3 years (13), and a subsequent study indicated that staged MV-PCI offered better clinical results than immediate MV-PCI in terms of a combined endpoint of cardiac death or MI for elderly patients with NSTE-ACS and MVD (14). Until now, there is only one randomized controlled trial exploring this question in the presence of NSTEMI and MVD. The SMILE (Impact of Different Treatment in Multivessel Non-ST Elevation Myocardial Infarction Patients) trial compared clinical prognosis between one-session strategy and staged strategy in setting of NSTEMI and MVD (11). That trial found that CR during the index intervention was related with a reduced composite risk of major adverse cardiovascular and cerebrovascular events, caused exclusively by reductions in target vessel revascularization. In contrast to SMILE, a propensity-matched analysis from Korea of 2,872 patients with NSTEMI and MVD found no differences between the immediate and staged MV-PCI strategies for MACE at 3 years (10). Therefore, reported findings in this field from randomized trials and observational studies have been somewhat inconsistent.

Our study compared long-term clinical outcomes between the immediate intervention strategy and the out-of-hospital staged intervention strategy, and investigated which patients were better suited for the revascularization strategy. In our study, the raw comparisons showed that staged PCI reduced the incidences of MACE and repeat revascularization significantly. Propensity-matched and multivariate Cox regression analyses found that the incidences of primary and secondary endpoints were comparable between the two groups. However, a definite separation in the Kaplan-Meier curve of MACE between the two strategies was observed, suggesting a potentially beneficial trend of the staged strategy in the reduction of MACE. The main reason for the insignificant statistical results may be the small sample size. Furthermore, given the heterogeneity of pathophysiology in the setting of NSTEMI and MVD, not all patients are suitable for staged MV-PCI strategy. Patient-, diseased-, and coronary lesion-based revascularization approaches should be performed to achieve personalized and precise medical treatment. Thus, we conducted subsequent subgroup analyses and found that there was a statistically lower risk of MACE for staged strategy in with the presence of complex coronary disease. These results are largely discordant with those of the SMILE trial and the previous Korean study. The main reason for this may be the different inclusion and exclusion criteria used. The randomized trial and previous observational studies of immediate strategy vs. staged strategy have focused on the patients who received staged PCI at the index hospitalization (10, 11), but with the patients undergoing staged PCI approach at the second admission excluded. The present study enrolled patients treated with staged MV-PCI < 60 days after discharge, in which the prolonged interval between index- and second-stage PCI could further reduce the drawbacks of immediate PCI. Therefore, the superiority of staged PCI strategy in particular subgroups should be validated.

The choice of strategy not only influences the efficacy and patient safety but also costs and reimbursements. Staged MV-PCI increases patient medical costs compared with immediate MV-PCI (30, 32). National insurance committees across most countries, including China, tend to deprecate staged PCI approaches. As such, cardiologists must weigh the economic disadvantages of staged MV-PCI against possible prognostic benefits. However, approximately half of the population who underwent MV-PCI in the present study chose staged strategy, which contrasted with the 21.2% rate seen in the Korean study (10). There is an urgent need to offer robust evidence justifying the reasonableness of the extra cost of staged MV-PCI. The present study demonstrated that a staged intervention may accord with the patients’ best interest, which may give right causes to promote staged intervention in the setting of NSTEMI and MVD. However, this was an observational study with inevitable limitations; large-scale, adequately powered trials comparing the two strategies are needed.

This study has several limitations. First, as this was an observational study, selection bias was inevitable. Although we used various analytical methods to minimize potential confounders, unmeasured variables could not be excluded entirely. Second, owing to the retrospective nature of this study and long-term follow-up, loss to follow-up was inevitable. The rate of loss to follow-up was 15.1% in our study, which met the requirement of < 20% in a retrospective cohort study. Third, the small sample size limited the statistical power of the present study. A clear benefit of staged MV-PCI may be demonstrated in large-scale studies. Fourth, our analysis was performed at a provincial center for cardiovascular diseases. The results of this single-center study may not be generalizable to all countries and regions. Therefore, large prospective randomized trials examining the three intervention strategies in NSTEMI patients with MVD are required. Fifth, intravascular imaging and fractional flow reserve (FFR) have been considered potentially valuable tools for characterizing and evaluating non-culprit lesions, although published evidence is somewhat lacking. The number of patients enrolled in our study who underwent FFR or intravascular imaging was small. The extent of this influence in the setting of the three strategies for NSTEMI patients with MVD is unclear, and more data in this area are required. Sixth, data on contrast media-induced nephropathy (CIN), radiation dose, procedure duration, and cost were not collected. Seventh, owing to the limited sample of patients treated with staged intervention at index admission, we were unable to further evaluate the efficacy of in-hospital staged MV-PCI.

## Conclusion

In hemodynamically stable patients with NSTEMI and MVD, MV-PCI reduced cardiovascular events compared with CL-PCI. Moreover, out-of-hospital MV-PCI seemed to be superior to immediate MV-PCI, especially for patients with complex coronary disease. These findings warrant verification in large, prospective, randomized trials.

## Data availability statement

The raw data supporting the conclusions of this article will be made available by the authors, without undue reservation.

## Ethics statement

The studies involving human participants were reviewed and approved by the Ethics Committee of Tianjin Chest Hospital. Written informed consent for participation was not required for this study in accordance with the national legislation and the institutional requirements.

## Author contributions

CW, LW, and HC conceived and designed the study. CW, JL, JZ, YH, CH, RX, and JW collected the clinical data. CW and LW conducted the statistical analyses and wrote the manuscript. CL, WL, TL, AW, WQ, and DJ consulted and supplemented the relevant information. LW and HC revised the manuscript and supervised the study. All authors contributed to the article revision, read, and approved the submitted version.
