# Supplementary material for: Culprit vessel vs. immediate multivessel vs. out-of-hospital staged intervention for patients with non-ST-segment elevation myocardial infarction and multivessel disease
Source: Front Cardiovasc Med. 2022 Nov 23;9:1033475. doi: 10.3389/fcvm.2022.1033475 (PMC9726786; doi:10.3389/fcvm.2022.1033475)
Supplement: Supplementary file 1 [file Data_Sheet_1.docx]

**Supplemental Material**

Culprit vessel vs. immediate multivessel vs. out-of-hospital staged intervention for patients with non-ST-segment elevation myocardial infarction and multivessel disease

Chen Wang^1,2^, Jiachun Lang^1,2^, Jingxia Zhang^2^, Yuecheng Hu^2^, Chuyi Han^1,2^, Rongdi Xu^1,2^, Jikun Wu^1,2^, Chunwei Liu^2^, Wenyu Li^2^, Tingting Li^2^, Ao Wei^2^, Wei Qi^2^, Dongxia Jin^2^, Hongliang Cong^1,2*^, Le Wang^2*^

**Supplemental Table 1**

|  | **Unadjusted** | | | **Propensity score adjusted** | | |
| --- | --- | --- | --- | --- | --- | --- |
|  | **CL-PCI**  **(N=330)** | **Immediate MV-PCI**  **(N=295)** | ***P* value** | **CL-PCI**  **(N=243)** | **Immediate MV-PCI**  **(N=243)** | ***P* value** |
| Age > 65 years | 148 (44.8%) | 125 (42.4%) | 0.533 | 106 (43.6%) | 105 (43.2%) | 0.927 |
| Male | 235 (71.2%) | 219 (74.2%) | 0.397 | 178 (73.3%) | 176 (72.4%) | 0.838 |
| Killip class Ⅱ-Ⅲ | 26 (7.9%) | 25 (8.5%) | 0.786 | 18 (7.4%) | 23 (9.5%) | 0.414 |
| Grace score > 140 | 98 (29.7%) | 92 (31.2%) | 0.686 | 75 (30.9%) | 75 (30.9%) | NA |
| Past medical history |  |  |  |  |  |  |
| Hypertension | 234 (70.9%) | 204 (69.4%) | 0.678 | 167 (68.7%) | 173 (71.2%) | 0.553 |
| Diabetes mellitus | 111 (33.6%) | 113 (38.3%) | 0.224 | 83 (34.2%) | 86 (35.4%) | 0.775 |
| Prior MI | 33 (10.0%) | 31 (10.5%) | 0.834 | 24 (9.9%) | 25 (10.3%) | 0.880 |
| Prior PCI | 41 (12.4%) | 33 (11.2%) | 0.644 | 29 (11.9%) | 29 (11.9%) | NA |
| Prior stroke | 61 (18.5%) | 64 (21.7%) | 0.317 | 47 (19.3%) | 46 (18.9%) | 0.908 |
| Current smoker | 148 (44.8%) | 139 (47.3%) | 0.543 | 110 (45.3%) | 112 (46.1%) | 0.855 |
| LVEF < 50% | 79 (23.9%) | 80 (27.1%) | 0.362 | 65 (26.7%) | 63 (25.9%) | 0.837 |
| Laboratory findings |  |  |  |  |  |  |
| Peak level of troponin, ng/ml | 0.6 (0.2-1.2) | 0.5 (0.2-1.1) | 0.447 | 0.6 (0.2-1.2) | 0.5 (0.2-1.1) | 0.351 |
| Peak level of CK-MB, ng/ml | 28.0 (17.0-60.0) | 30.0 (17.0-53.6) | 0.889 | 28.0 (17.0-55.0) | 29.0 (18.0-53.6) | 0.936 |
| eGFR < 60 ml/min/1.73 m² | 52 (15.8%) | 37 (12.5%) | 0.251 | 31 (12.8%) | 32 (13.2%) | 0.893 |
| Medications at discharge |  |  |  |  |  |  |
| Aspirin | 321 (97.3%) | 294 (99.7%) | 0.022 | 242 (99.6%) | 242 (99.6%) | NA |
| P_2_Y_12_ inhibitor | 330 (100.0%) | 295 (100.0%) | NA | 243 (100.0%) | 243 (100.0%) | NA |
| ACEI or ARB or ARNI | 206 (62.4%) | 198 (67.1%) | 0.220 | 162 (66.7%) | 163 (67.1%) | 0.923 |
| Beta-blocker | 225 (68.2%) | 223 (75.6%) | 0.040 | 182 (74.9%) | 179 (73.7%) | 0.756 |
| Statin | 321 (97.3%) | 277 (93.9%) | 0.038 | 235 (96.7%) | 233 (95.9%) | 0.631 |

MI, myocardial infarction; PCI, percutaneous coronary intervention; LVEF, left ventricular ejection fraction; SBP, systolic blood pressure; HR, heart rate; CK-MB, creatine kinase-myocardial band; eGFR, estimated glomerular filtration rate; ACEI, angiotensin-converting enzyme inhibitor; ARB, angiotensin-II receptor blocker; ARNI, angiotensin receptor neprilysin inhibitor.

**Supplemental Table 2**

|  | **Unadjusted** | | | **Propensity score adjusted** | | |
| --- | --- | --- | --- | --- | --- | --- |
|  | **CL-PCI**  **(N=330)** | **Immediate MV-PCI**  **(N=295)** | ***P* value** | **CL-PCI**  **(N=243)** | **Immediate MV-PCI**  **(N=243)** | ***P* value** |
| Culprit lesion profiles |  |  |  |  |  |  |
| Location of culprit lesions | - | - | 0.001 | - | - | 0.788 |
| Left main coronary artery | 12 (3.6%) | 21 (7.1%) | - | 11 (4.5%) | 14 (5.8%) | - |
| Left anterior descending artery | 113 (34.2%) | 124 (42.0%) | - | 104 (42.8%) | 96 (39.5%) | - |
| Left circumflex artery | 111 (33.6%) | 108 (36.7%) | - | 84 (34.6%) | 93 (38.4%) | - |
| Right coronary artery | 110 (33.3%) | 60 (20.3%) | **-** | 57 (23.5%) | 53 (21.8%) | - |
| ACC/AHA lesion type |  |  |  |  |  |  |
| B_2_/C | 300 (90.9%) | 246 (83.4%) | 0.005 | 215 (88.5%) | 213 (87.7%) | 0.780 |
| Overall-lesion profiles |  |  |  |  |  |  |
| Left main disease | 34 (10.3%) | 42 (14.2%) | 0.133 | 28 (11.5%) | 24 (9.9%) | 0.557 |
| Triple vessel disease | 245 (74.2%) | 208 (71.0%) | 0.363 | 176 (72.4%) | 175 (72.0%) | 0.919 |
| SYNTAX score > 22 | 88 (26.7%) | 67 (22.7%) | 0.253 | 56 (23.0%) | 56 (23.0%) | NA |
| Procedural characteristics |  |  |  |  |  |  |
| Radial artery access | 255 (77.3%) | 232 (78.6%) | 0.680 | 188 (77.4%) | 191 (78.6%) | 0.743 |
| IVUS guide PCI | 7 (2.1%) | 7 (2.4%) | 0.832 | 7 (2.9%) | 5 (2.1%) | 0.559 |
| IABP | 8 (2.4%) | 8 (2.7%) | 0.820 | 7 (2.9%) | 4 (1.6%) | 0.360 |

ACC/AHA, American College of Cardiology/American Heart Association; SYNTAX, Synergy between PCI with Taxus and Cardiac Surgery; IVUS, intravascular ultrasound; PCI, percutaneous coronary intervention; IABP, intra-aortic balloon pump.

**Supplemental Table 3**

|  | **Unadjusted** | | | **Propensity score adjusted** | | |
| --- | --- | --- | --- | --- | --- | --- |
|  | **CL-PCI**  **(N=330)** | **Staged MV-PCI**  **(N=318)** | ***P* value** | **CL-PCI**  **(N=246)** | **Staged MV-PCI**  **(N=246)** | ***P* value** |
| Age > 65 years | 148 (44.8%) | 124 (39.0%) | 0.131 | 101 (41.1%) | 106 (43.1%) | 0.648 |
| Male | 235 (71.2%) | 234 (73.6%) | 0.499 | 180 (73.2%) | 183 (74.4%) | 0.758 |
| Killip class Ⅱ-Ⅲ | 26 (7.9%) | 13 (4.1%) | 0.043 | 14 (5.7%) | 11 (4.5%) | 0.538 |
| Grace score > 140 | 98 (29.7%) | 71 (22.3%) | 0.033 | 64 (26.0%) | 60 (24.4%) | 0.678 |
| Past medical history |  |  |  |  |  |  |
| Hypertension | 234 (70.9%) | 206 (64.8%) | 0.095 | 167 (67.9%) | 166 (67.5%) | 0.923 |
| Diabetes mellitus | 111 (33.6%) | 121 (38.1%) | 0.241 | 83 (33.7%) | 86 (35.0%) | 0.776 |
| Prior MI | 33 (10.0%) | 32 (10.1%) | 0.979 | 23 (9.3%) | 23 (9.3%) | NA |
| Prior PCI | 41 (12.4%) | 25 (7.9%) | 0.055 | 23 (9.3%) | 25 (10.2%) | 0.761 |
| Prior stroke | 61 (18.5%) | 54 (17.0%) | 0.616 | 39 (15.9%) | 44 (17.9%) | 0.547 |
| Current smoker | 148 (44.8%) | 157 (49.4%) | 0.249 | 117 (47.6%) | 115 (46.7%) | 0.857 |
| LVEF < 50% | 79 (23.9%) | 60 (18.9%) | 0.116 | 55 (22.4%) | 45 (18.3%) | 0.263 |
| Laboratory findings |  |  |  |  |  |  |
| Peak level of troponin, ng/ml | 0.6 (0.2-1.2) | 0.6 (0.3-1.3) | 0.249 | 0.6 (0.2-1.3) | 0.6 (0.3-1.3) | 0.407 |
| Peak level of CK-MB, ng/ml | 28.0 (17.0-60.0) | 33.0 (19.0-61.3) | 0.072 | 32.0 (18.8-64.3) | 34.0 (19.0-60.0) | 0.665 |
| eGFR < 60 ml/min/1.73 m² | 52 (15.8%) | 24 (7.5%) | 0.001 | 24 (9.8%) | 24 (9.8%) | NA |
| Medications at discharge |  |  |  |  |  |  |
| Aspirin | 321 (97.3%) | 314 (98.7%) | 0.182 | 243 (98.8%) | 243 (98.8%) | NA |
| P_2_Y_12_ inhibitor | 330 (100.0%) | 318 (100.0%) | NA | 246 (100%) | 246 (100%) | NA |
| ACEI or ARB or ARNI | 206 (62.4%) | 200 (62.9%) | 0.902 | 148 (60.2%) | 152 (61.8%) | 0.712 |
| Beta-blocker | 225 (68.2%) | 234 (73.6%) | 0.130 | 175 (71.1%) | 170 (69.1%) | 0.622 |
| Statin | 321 (97.3%) | 302 (95.0%) | 0.128 | 238 (96.7%) | 235 (95.5%) | 0.483 |

MI, myocardial infarction; PCI, percutaneous coronary intervention; LVEF, left ventricular ejection fraction; SBP, systolic blood pressure; HR, heart rate; CK-MB, creatine kinase-myocardial band; eGFR, estimated glomerular filtration rate; ACEI, angiotensin-converting enzyme inhibitor; ARB, angiotensin-II receptor blocker; ARNI, angiotensin receptor neprilysin inhibitor.

**Supplemental Table 4**

|  | **Unadjusted** | | | **Propensity score adjusted** | | |
| --- | --- | --- | --- | --- | --- | --- |
|  | **CL-PCI**  **(N=330)** | **Staged MV-PCI**  **(N=318)** | ***P* value** | **CL-PCI**  **(N=246)** | **Staged MV-PCI**  **(N=246)** | ***P* value** |
| Culprit lesion profiles |  |  |  |  |  |  |
| Location of culprit lesions | - | - | 0.026 | - | - | 0.833 |
| Left main coronary artery | 12 (3.6%) | 4 (1.3%) | - | 4 (1.6%) | 4 (1.6%) | - |
| Left anterior descending artery | 113 (34.2%) | 88 (27.7%) | - | 74 (30.1%) | 82 (33.3%) | - |
| Left circumflex artery | 111 (33.6%) | 114 (35.8%) | - | 83 (33.7%) | 87 (35.4%) | - |
| Right coronary artery | 110 (33.3%) | 146 (45.9%) | - | 92 (37.4%) | 100 (40.7%) | - |
| ACC/AHA lesion type |  |  |  |  |  |  |
| B_2_/C | 300 (90.9%) | 308 (96.9%) | 0.002 | 237 (96.3%) | 236 (95.9%) | 0.815 |
| Overall-lesion profiles |  |  |  |  |  |  |
| Left main disease | 34 (10.3%) | 21 (6.6%) | 0.091 | 23 (9.3%) | 20 (8.1%) | 0.632 |
| Triple vessel disease | 245 (74.2%) | 249 (78.3%) | 0.225 | 189 (76.8%) | 189 (76.8%) | NA |
| SYNTAX score > 22 | 88 (26.7%) | 91 (28.6%) | 0.579 | 66 (26.8%) | 65 (26.4%) | 0.919 |
| Procedural characteristics |  |  |  |  |  |  |
| Radial artery access | 255 (77.3%) | 262 (82.4%) | 0.105 | 193 (78.5%) | 202 (82.1%) | 0.308 |
| IVUS guide PCI | 7 (2.1%) | 5 (1.6%) | 0.604 | 5 (2.0%) | 5 (2.0%) | NA |
| IABP | 8 (2.4%) | 4 (1.3%) | 0.271 | 5 (2.0%) | 2 (0.8%) | 0.450 |

ACC/AHA, American College of Cardiology/American Heart Association; SYNTAX, Synergy between PCI with Taxus and Cardiac Surgery; IVUS, intravascular ultrasound; PCI, percutaneous coronary intervention; IABP, intra-aortic balloon pump.

**Supplemental Table 5**

|  | **Unadjusted** | | | **Propensity score adjusted** | | |
| --- | --- | --- | --- | --- | --- | --- |
|  | **Immediate MV-PCI**  **(N=295)** | **Staged MV-PCI**  **(N=318)** | ***P* value** | **Immediate MV-PCI**  **(N=201)** | **Staged MV-PCI**  **(N=201)** | ***P* value** |
| Age > 65 years | 125 (42.4%) | 124 (39.0%) | 0.395 | 87 (43.3%) | 81 (40.3%) | 0.544 |
| Male | 219 (74.2%) | 234 (73.6%) | 0.854 | 149 (74.1%) | 146 (72.6%) | 0.735 |
| Killip class Ⅱ-Ⅲ | 25 (8.5%) | 13 (4.1%) | 0.024 | 13 (6.5%) | 9 (4.5%) | 0.380 |
| Grace score > 140 | 92 (31.2%) | 71 (22.3%) | 0.013 | 62 (30.8%) | 54 (26.9%) | 0.379 |
| Past medical history |  |  |  |  |  |  |
| Hypertension | 204 (69.4%) | 206 (64.8%) | 0.226 | 140 (69.7%) | 141 (70.1%) | 0.913 |
| Diabetes mellitus | 113 (38.3%) | 121 (38.1%) | 0.948 | 79 (39.3%) | 74 (36.8%) | 0.608 |
| Prior MI | 31 (10.5%) | 32 (10.1%) | 0.856 | 19 (9.5%) | 18 (9.0%) | 0.863 |
| Prior PCI | 33 (11.2%) | 25 (7.9%) | 0.156 | 18 (9.0%) | 19 (9.5%) | 0.863 |
| Prior stroke | 64 (21.7%) | 54 (17.0%) | 0.139 | 37 (18.4%) | 38 (18.9%) | 0.898 |
| Current smoker | 139 (47.3%) | 157 (49.4%) | 0.605 | 96 (47.8%) | 95 (47.3%) | 0.920 |
| LVEF < 50% | 80 (27.1%) | 60 (18.9%) | 0.015 | 48 (23.9%) | 45 (22.4%) | 0.723 |
| Laboratory findings |  |  |  |  |  |  |
| Peak level of troponin, ng/ml | 0.5 (0.2-1.1) | 0.6 (0.3-1.3) | 0.062 | 0.5 (0.2-1.2) | 0.6 (0.3-1.2) | 0.359 |
| Peak level of CK-MB, ng/ml | 30.0 (17.0-53.6) | 33.0 (19.0-61.3) | 0.054 | 30.0 (19.0-53.6) | 32.0 (19.0-58.8) | 0.610 |
| eGFR < 60 ml/min/1.73 m² | 37 (12.5%) | 24 (7.5%) | 0.039 | 19 (9.5%) | 19 (9.5%) | NA |
| Medications at discharge |  |  |  |  |  |  |
| Aspirin | 294 (99.7%) | 314 (98.7%) | 0.375 | 201 (100.0%) | 201 (100.0%) | NA |
| P_2_Y_12_ inhibitor | 295 (100%) | 318 (100%) | NA | 201 (100.0%) | 201 (100.0%) | NA |
| ACEI or ARB or ARNI | 198 (67.1%) | 200 (62.9%) | 0.273 | 133 (66.2%) | 134 (66.7%) | 0.916 |
| Beta-blocker | 223 (75.6%) | 234 (73.6%) | 0.568 | 156 (77.6%) | 152 (75.6%) | 0.637 |
| Statin | 277 (93.9%) | 302 (95.0%) | 0.563 | 188 (93.5%) | 189 (94.0%) | 0.836 |

MI, myocardial infarction; PCI, percutaneous coronary intervention; LVEF, left ventricular ejection fraction; SBP, systolic blood pressure; HR, heart rate; CK-MB, creatine kinase-myocardial band; eGFR, estimated glomerular filtration rate; ACEI, angiotensin-converting enzyme inhibitor; ARB, angiotensin-II receptor blocker; ARNI, angiotensin receptor neprilysin inhibitor.

**Supplemental Table 6**

|  | **Unadjusted** | | | **Propensity score adjusted** | | |
| --- | --- | --- | --- | --- | --- | --- |
|  | **Immediate MV-PCI**  **(N=295)** | **Staged MV-PCI**  **(N=318)** | ***P* value** | **Immediate MV-PCI**  **(N=201)** | **Staged MV-PCI**  **(N=201)** | ***P* value** |
| Culprit lesion profiles |  |  |  |  |  |  |
| Location of culprit lesions | - | - | < 0.001 | - | - | 0.679 |
| Left main coronary artery | 21 (7.1%) | 4 (1.3%) | - | 8 (4.0%) | 4 (2.0%) | - |
| Left anterior descending artery | 124 (42.0%) | 88 (27.7%) | - | 73 (36.3%) | 79 (39.3%) | - |
| Left circumflex artery | 108 (36.7%) | 114 (35.8%) | - | 80 (39.8%) | 83 (41.3%) | - |
| Right coronary artery | 60 (20.3%) | 146 (45.9%) | - | 49 (24.4%) | 60 (29.9%) | - |
| ACC/AHA lesion type |  |  |  |  |  |  |
| B_2_/C | 246 (83.4%) | 308 (96.9%) | < 0.001 | 193 (96.0%) | 191 (95.0%) | 0.630 |
| Overall-lesion profiles |  |  |  |  |  |  |
| Left main disease | 42 (14.2%) | 21 (6.6%) | 0.002 | 16 (8.0%) | 14 (7.0%) | 0.704 |
| Triple vessel disease | 208 (71.0%) | 249 (78.3%) | 0.038 | 153 (76.1%) | 151 (75.1%) | 0.816 |
| SYNTAX score > 22 | 67 (22.7%) | 91 (28.6%) | 0.095 | 47 (23.4%) | 42 (20.9%) | 0.548 |
| Procedural characteristics |  |  |  |  |  |  |
| Radial artery access | 232 (78.6%) | 262 (82.4%) | 0.241 | 159 (79.1%) | 161 (80.1%) | 0.804 |
| IVUS guide PCI | 7 (2.4%) | 5 (1.6%) | 0.475 | 5 (2.5%) | 2 (1.0%) | 0.449 |
| IABP | 8 (2.7%) | 4 (1.3%) | 0.194 | 4 (2.0%) | 2 (1.0%) | 0.685 |

ACC/AHA, American College of Cardiology/American Heart Association; SYNTAX, Synergy between PCI with Taxus and Cardiac Surgery; IVUS, intravascular ultrasound; PCI, percutaneous coronary intervention; IABP, intra-aortic balloon pump.
